# On the Optimization of Carob Seed Peel Extraction Using Aqueous-Based Acidic Systems

**DOI:** 10.3390/molecules30071397

**Published:** 2025-03-21

**Authors:** Bruno Medronho, Oumaima Boutoub, Hugo Duarte, Maria José Aliaño-González, Rui Guerra, António Brázio, Sandra Gonçalves, Anabela Romano

**Affiliations:** 1 MED-Mediterranean Institute for Agriculture, Environment and Development, CHANGE-Global Change and Sustainability Institute, Faculdade de Ciências e Tecnologia, Universidade do Algarve, Campus de Gambelas, Ed. 8, 8005-139 Faro, Portugal; boutoub.oumaima@gmail.com (O.B.); uc49007@uc.pt (H.D.); mariajose.aliano@gm.uca.es (M.J.A.-G.); 2 FSCN, Surface and Colloid Engineering, Mid Sweden University, SE-851 70 Sundsvall, Sweden; 3 Pólo II–R. Silvio Lima, Department of Chemical Engineering, CERES, University of Coimbra, 3030-790 Coimbra, Portugal; 4 Analytical Chemistry Department, University of Cádiz, 11510 Puerto Real, Spain; 5 Center for Electronics, Optoelectronics and Telecommunications (CEOT), Faculdade de Ciências e Tecnologia, Universidade do Algarve, 8005-139 Faro, Portugal; rguerra@ualg.pt (R.G.); ambrazio@ualg.pt (A.B.); 6 Departamento de Física, Faculdade de Ciências e Tecnologia, Universidade do Algarve, Campus de Gambelas, 8005-139 Faro, Portugal

**Keywords:** carob seeds, husk extraction, design of experiments, methanesulfonic acid, diffusive reflectance spectroscopy

## Abstract

Carob fruit utilization remains limited, with most of their commercial value derived from locust bean gum, which is obtained from seed endosperm. Efficient extraction requires dehusking, which is traditionally performed under harsh conditions. This study aims to develop and optimize a milder, more sustainable dehusking method while preserving seed quality for industrial applications. Various aqueous-based solvents were tested, leading to the selection of metanesulfonic acid (CH_4_O_3_S). A Box–Behnken design with response surface methodology optimized the process, using husk removal efficiency as the response variable. The optimized conditions were 24.5 g of seeds treated in 50 mL of a solvent mixture (41% CH_4_O_3_S and 59% H_2_SO_4_) at 90 °C for 10 min, followed by washing by 5 min with water (87 mL). The treated seeds were analyzed using colorimetry assays and diffusive reflectance spectroscopy and benchmarked against both pristine and dehusked seeds from a local company. The resulting seeds remained morphologically intact and exhibited appealing color characteristics comparable to commercial samples. The optimized method ensured intact seed morphology and color characteristics comparable to commercial standards, offering a viable alternative to conventional H_2_SO_4_-based dehusking. Furthermore, this study also highlights for the first time the effectiveness of diffusive reflectance spectroscopy as a rapid and straightforward tool for assessing the dehusking process.

## 1. Introduction

The carob tree (*Ceratonia siliqua* L.), which is native to the Middle East, is cultivated throughout the Mediterranean basin, particularly in Tunisia, Egypt, Cyprus, Greece, Italy, Spain, Portugal, and Morocco [1]. Although this species is of great economic and environmental significance in this region, most of these countries are underutilizing this natural resource, both within the industrial sector and among the general population. Given the nutritional value of all the plant’s edible parts, the potential of this resource deserves greater recognition [2]. The fruit, an indehiscent pod consisting of pulp and seeds, is the primary source of carob tree industrial value. The pulp, which represents the main part of the fruit, is rich in sugar and is extensively used in the agri-food industry as a substitute for cocoa in cakes and other baked goods, in the production of syrup and molasses [3,4,5]. As it also contains dietary fiber and a variety of bioactive compounds, it has potential application in pharmaceutical products. Carob seeds are composed of three main parts: the shell, the embryo, and the endosperm [6]. The shell (also known as peel or husk) is a tough brown covering that accounts for roughly 30–35% of the seed’s dry weight. The embryo, located at the seed’s center, comprises approximately 15–30% of the dry weight and is rich in protein, enzymes, dietary fibers, minerals, and polyphenols. The endosperm, located between the rootlet and the seed husk, constitutes approximately 40–50% of the seed’s total weight. The endosperm of carob seeds is primarily used to produce locust bean gum (LBG), a natural galactomannan-based biopolymer that is extensively employed in food formulations as E410. This substance is highly prized for its superior thickening, stabilizing, emulsifying, and gelling properties [7]. These beneficial characteristics, along with its non-toxic nature, make LBG suitable for utilization in diverse sectors, including textiles, pharmaceuticals, biomedical fields, cosmetics, nutrition, and food science [6,8]. The production of carob gum commences with the removal of the hard shell from the seed, a process that can be accomplished through thermomechanical or chemical treatments [6].

In the water-dehulling method, carob seeds are immersed in boiling water for approximately one hour. Following this, the seeds are removed and washed, and the husk is easily broken and manually separated from the endosperm, yielding yellowish gum. By contrast, the acid-peeling method involves treating carob seeds with sulfuric acid at high temperatures to carbonize the seed coat. The residual fragments of the husk are then eliminated through washing and brushing, resulting in a white gum. A comparison of these two approaches reveals that the acid-peeling process produced LBG with superior thickening properties, as evidenced by its higher galactomannan content, enhanced solubility at high temperatures, larger molecular size, and greater intrinsic viscosity [9]. An alternative method involves subjecting carob kernels to roasting in a rotating furnace, a process which detaches the husk. However, this results in LBG with a darker color due to the high temperatures involved. Elevated processing temperatures can reduce the gum’s ability to achieve high viscosities, thereby diminishing its overall value [10].

Industrial-scale removal of carob seed husks typically employs H_2_SO_4_ at elevated temperatures to carbonize the seed husk. Specific temperature and concentration parameters are often proprietary and not extensively detailed in public literature. Whilst the conventional H_2_SO_4_-based dehusking process is effective, it is also harsh and polluting, giving rise to economic and environmental concerns due to its corrosiveness, oxidizing nature, and the potential formation of toxic SOₓ gases. Additionally, the effluent must undergo proper treatment to prevent contamination, and the process poses risks to workers. In addressing these challenges, this study explores the potential of aqueous-based acidic systems, including methanesulfonic acid (MSA), as a more sustainable alternative for carob seed peel extraction. The utilization of MSA over H_2_SO_4_ is advantageous due to its reduced toxicity, enhanced biodegradability, and diminished generation of hazardous byproducts, while maintaining robust acid strength and catalytic efficiency. In contrast to sulfuric acid, MSA does not generate SO_x_ emissions and has exhibited superior performance in biomass processing applications, rendering it a promising candidate for the food and biopolymer industries. To remove any residual fragments, the treated seeds must then be thoroughly washed and brushed. Following grinding and separation from the germ, the resulting LBG is a white powder with high viscosity [9]. However, data suggest that this harsh treatment may alter the native physicochemical properties of the endosperm, potentially impacting its quality. Therefore, to optimize the utilization of carob seeds, it is essential to develop more sustainable extraction methods. In this regard, the primary objective of this study is to assess the efficacy of various organic and inorganic aqueous-based acidic solvents and to determine the optimal extraction conditions through the implementation of a systematic experimental design. The present study represents a pioneering endeavor in the field, with its integration of diffuse reflectance spectroscopy coupled with chemometric analysis. This innovative approach enables the quantitative evaluation of extraction efficiency and the optimization of process conditions. This analytical approach provides a more precise and systematic assessment of the impact of different solvents, moving beyond traditional visual inspection and colorimetry. The adoption of this innovative methodology is expected to enhance the quality of LBG and establish a scalable, eco-friendly, and industry-relevant approach to carob seed processing. This will ultimately contribute to the sustainable valorization of this underutilized resource.

## 2. Results and Discussion

### 2.1. Initial Solvent Screening

**Figure 1:**
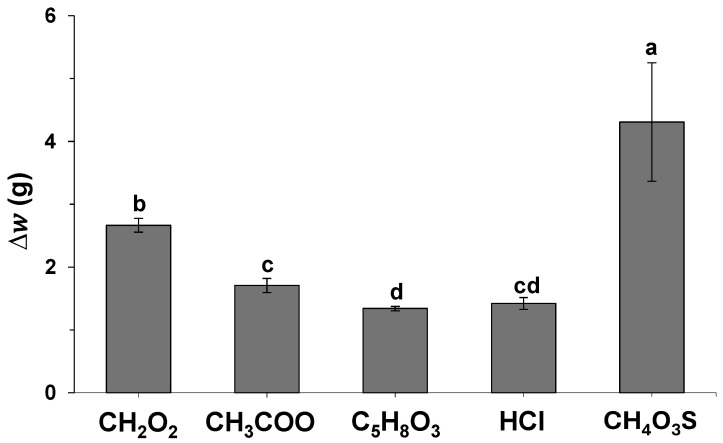
Difference in weight (∆w) of carob seeds after treatment with different solvent mixtures containing 80% (*v*/*v*) selected solvent: formic acid (CH_2_O_2_), acetic acid (CH_3_COOH), levulinic acid (C_5_H_8_O_3_), hydrochloric acid (HCl) and methanesulfonic acid (CH_4_O_3_S) and 20% H_2_SO_4_. Different letters on top of each bar represent significant differences at 95% confidence.

The influence of different acidic systems on carob seed skin extraction was first evaluated at room temperature using five selected solvents: formic acid, acetic acid, levulinic acid, hydrochloric acid, and methanesulfonic acid. These solvents were selected based on the group’s expertise in using them for biomass treatment and lignocellulose fractionation [11,12,13]. The performance of these solvents was found to be modest when used alone, and, therefore, they were combined with H_2_SO_4_ at different volume fractions. The solvent systems that were selected were then placed in contact with the seeds for 960 min at 25 °C, at a mixing ratio of 1:5 (i.e., H_2_SO_4_: alternative solvent). The seeds were removed from each solvent and washed with water. The weight lost resulting from the peel extraction was estimated using Equation (1). All experiments were carried out in triplicate, and the average weight difference, ∆w, is plotted in Figure 1.

An ANOVA with 95% confidence was performed to evaluate the differences according to the solvent used. A *p*-value of 2.44 × 10^−7^ was obtained, significantly lower than 0.05, which implies a significant influence of the nature of the solvent in the extraction process. In this regard, it was observed that methanesulfonic acid is the best solvent tested with a weight difference of approximately 4.3 g (i.e., 17% weight reduction). Conversely, levulinic acid presented the lowest impact in husk extraction with an observed weight difference of approximately 1 g (i.e., 5% weight reduction).

### 2.2. Extraction Optimization with BBD-RSM

**Figure 2:**
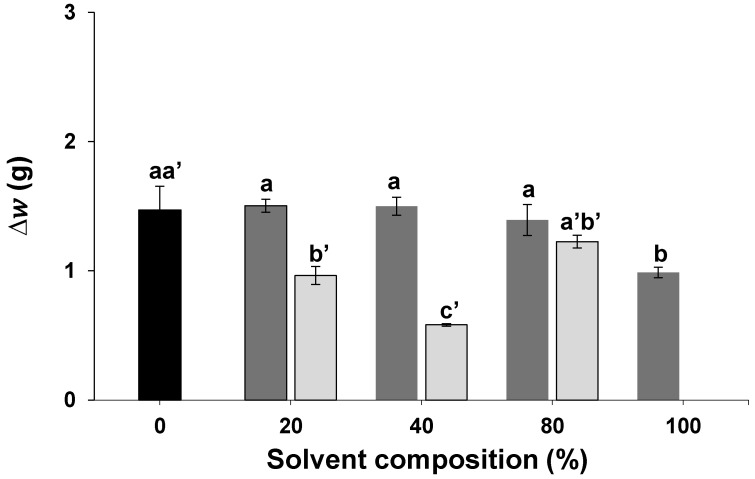
Difference in weight (∆w) of carob seeds after treatment with CH_4_O_3_S/H_2_SO_4_ (dark gray) and CH_2_O_2_/H_2_SO_4_ (light gray) solvents with different volume ratios. The black bar represents the pure H_2_SO_4_ solvent. Different letters on top of each bar represent significant differences at 95% confidence.

After identifying the most promising solvent systems, the next step involved the development of a design of experiments (DOE) to evaluate the variables that most significantly influence husk extraction. A key aspect of the DOE was to assess and establish appropriate ranges for the variables under investigation. Initial extractions were conducted utilizing various ratios of CH_4_O_3_S to H_2_SO_4_ (i.e., 20, 40, 60, 80 and 100% (*v*/*v*)). The results from the previous assay indicated CH_2_O_2_ as the second most impactful solvent system after CH_4_O_3_S. Consequently, it was also selected for further optimization, as it is commonly employed in solid/liquid extractions [14,15]. The extraction conditions were set at 60 °C, and the extraction time was reduced to 30 min. The temperature and extraction time were selected based on preliminary assays and literature [9]. The average weight difference is plotted in Figure 2.

An ANOVA at the 95% confidence level was conducted to assess the impact of solvent percentage on extraction. The *p*-values for CH_2_O_2_and CH_4_O_3_S-based solvents were 2.13 × 10^−12^ and 3.39 × 10^−7^, respectively, indicating the influence of the percentage of solvent used. It was observed that replacing H_2_SO_4_ with increasing amounts of CH_2_O_2_ (20% and 40%) led to a decrease in solvent efficiency and, consequently, a reduction in the amount of seed peel extracted (e.g., the CH_2_O_2_/H_2_SO_4_ mixture containing 40% CH_2_O_2_ removed only approximately 40% of the seed peel compared to pure H_2_SO_4_). It is noteworthy that when the CH_2_O_2_ content was increased to 80% or higher, complete seed husk removal was attained; however, this also resulted in substantial degradation of several seeds. This explains the considerably larger error bars observed for these compositions.

In the case of CH_4_O_3_S/H_2_SO_4_ mixtures, up to 80% of H_2_SO_4_ could be replaced by CH_4_O_3_S without significantly compromising solvent efficiency (i.e., the CH_4_O_3_S/H_2_SO_4_ system containing 80% CH_4_O_3_S was still capable of removing approximately 95% of the seed husk). Under the tested conditions, pure CH_4_O_3_S was not as effective as the H_2_SO_4_ used by the company, but it still achieved around 67% seed husk removal.

Given the superior performance of CH_4_O_3_S-based solvents compared to CH_2_O_2_-based solvents, the focus was placed on the former. Furthermore, CH_4_O_3_S is considered a “green acid” due to its higher biodegradability and lower toxicity/corrosivity compared to mineral acids like H_2_SO_4_ [16,17]. Given its capacity to substitute up to 80% of H_2_SO_4_ without compromising solvent efficiency, CH_4_O_3_S emerges as a highly compelling alternative exhibiting a more favorable environmental profile than the use of H_2_SO_4_ alone.

After selecting CH_4_O_3_S, a BBD-RSM approach was employed to optimize seed husk extraction considering six variables: the solid–liquid ratio (i.e., grams of seeds in 50 mL solvent), temperature (ºC), the fraction of CH_4_O_3_S in the solvent mixture (%), the extraction time (min), the volume of water used for the seeds’ post-extraction wash (mL), and the washing time (min). The combination of all these variables resulted in a total of 54 experiments (Table 1), which were carried out randomly. The difference in seed weight (Equation (1)) was used as the response variable.

The results of the BBD-RSM study were utilized to develop a polynomial mathematical model to assess the influence of variables on seed peel extraction and to predict the optimal values of each variable for maximum extraction. The model evidenced an R^2^ coefficient of 0.833. Subsequently, a Durbin–Watson analysis was conducted to determine whether the order of analysis of the results influenced the development of the mathematical model. A *p*-value of 2.20 was obtained, which is significantly greater than 0.05, thereby indicating no influence from the order of analysis of the results. Subsequently, an ANOVA with 95% confidence was performed to evaluate the impact of the selected variables on the extraction process. The results obtained are presented in Table 2.

As observed in Table 2, the variables that had a significantly influential effect were the solid-to-liquid ratio (*p*-value = 0.0000), extraction temperature (*p*-value = 0.0013), and extraction time (*p*-value = 0.0057). The correlations between the solid-to-liquid ratio and extraction time (*p*-value = 0.0100), solid-to-liquid ratio and washing time (*p*-value = 0.0143), and extraction temperature and extraction time (*p*-value = 0.0193) were all found to have also *p*-values below 0.05.

**Figure 3:**
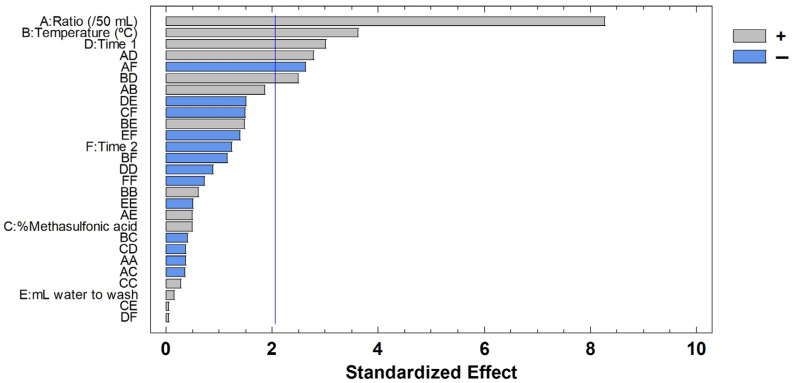
Pareto chart for seed husk removal after using BBD-RSM. The vertical line represents 95% significance.

In order to facilitate the visualization of the data, the results were graphically represented in a Pareto diagram (Figure 3), which confirms that most variables have a positive influence on seed husk removal. This indicates that as these variables approach the upper limit of the studied range, ∆*w* increases, resulting in greater seed husk extraction. However, the interaction between the solid-to-liquid ratio and washing time exhibited a negative effect.

**Figure 4:**
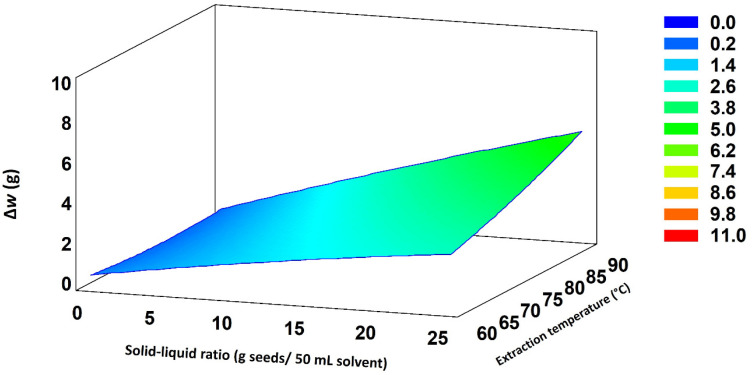
Response surface plot showing the combined effect of solid–liquid ratio and extraction temperature on seed husk. The warmer the color, the higher the amount of seed peel removed.

Since the solid–liquid ratio and extraction temperature were found to be the most influential variables, their influence was represented in a response surface diagram (Figure 4), using ∆w as the response variable. It can be observed that ∆w was greater when the values corresponding to both variables were in the upper limit of the range studied.

The determined coefficients for each of the selected variables are represented in Equation (2).

*Y* = −2.22 − 0.08 × X_1_ − 0.27 × X_2_ + 0.07 × X_3_ − 0.48 × X_4_+ 0.08 × X_5_ + 2.45 × X_6_ − 8.70 × 10^−4^ × X_1_^2^ + 0.003 × X_1_X_2_ − 3.56 × 10^−4^ × X_1_X_3_ + 0.02 × X_1_X_4_ + 5.99 × 10^−4^ × X_1_X_5_ − 0.03 × X_1_X_6_ + 8.13 × 10^−4^ × X_2_^2^ − 3.06 × 10^−4^ × X_2_X_3_ + 0.02 × X_2_X_4_ + 9.56 × 10^−4^ × X_2_X_5_ − 0.01 × X_2_X_6_ + 9.33× 10^−5^ × X_3_^2^ − 0.001 × X_3_X_4_ + 2.46 × 10^−5^ × X_3_X_5_ − 0.004 × X_3_X_6_ − 0.04 × X_4_^2^ − 0.008 × X_4_X_5_ + 0.002 × X_4_X_6_ − 2.42 × 10^−4^ × X_5_^2^ − 0.007 × X_5_X_6_ − 0.03 × X_6_^2^

Once the optimization method was completed, the optimal conditions for achieving the highest extraction yield were identified as follows: 24.5 g of the seeds in 50 mL of the solvent mixture containing 41% CH_4_O_3_S and 59% H_2_SO_4_, and extracted at 90 °C for 10 min. After extraction, seeds are removed and washed with 87 mL of water for 5 min.

**Figure 5:**
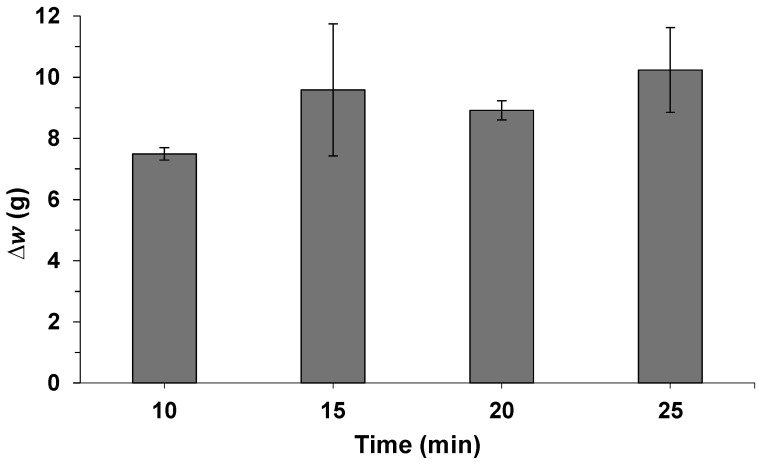
Difference in weight (∆w) of carob seeds after extraction under the optimal conditions determined, but with varying extraction times.

Despite the optimization described above, it was observed that the contact time of the solvent with the seeds (i.e., extraction time) reached its optimum value at the upper end of the range studied. Therefore, a kinetic study was further conducted to evaluate the influence of the extraction time. For this purpose, different extractions were performed under the determined optimal conditions but using different extraction times (i.e., 10, 15, 20, and 25 min). The ∆w is plotted in Figure 5.

At first glance, data suggest that ∆w increases with contact time. However, after performing a one-factor ANOVA, a *p*-value of 0.316 was obtained, indicating that there were no significant differences in ∆w for the extraction times evaluated. It is noteworthy that longer extraction times imply higher energy consumption, and as green chemistry principles underline the current research, 10 min was chosen as the extraction time. Lower times were not evaluated because 10 min was considered the minimum time to reach equilibrium.

The optimized extraction conditions demonstrate a promising balance between efficiency, feasibility, and economic viability, making them potentially suitable for large-scale applications. The use of 41% CH_4_O_3_S and 59% H_2_SO_4_ results in a substantial reduction in environmental hazards when compared to conventional methods that rely exclusively on concentrated H_2_SO_4_. As previously referenced, CH_4_O_3_S is characterized by its biodegradability, non-volatility, and reduced corrosivity, thereby enhancing handling safety and minimizing hazardous gas emissions (e.g., SO_x_). The short extraction time (10 min) and moderate temperature (90 °C) contribute to energy efficiency, reducing operational costs. The post-extraction washing step further simplifies purification while minimizing chemical waste. The established industrial use, recyclability, and lower environmental impact of methanesulfonic acid make it a promising alternative for sustainable carob husk extraction. Future studies should explore process scale-up, cost analysis, and solvent recovery strategies to enhance economic feasibility while maintaining high extraction efficiency.

### 2.3. Repeatability and Intermediate Precision

A repeatability and intermediate precision study was conducted to evaluate the robustness of the developed method. To assess repeatability, nine experiments were conducted under optimal conditions on the same day. For intermediate precision, nine experiments were performed over three consecutive days (*n* = 3 + 3 + 3). The coefficient of variation was selected as the statistical parameter to assess variation in this experiment.

As shown in Table 3, the coefficients of variation were consistently below 5% in both cases, thereby confirming the method’s repeatability and precision. The data thus support the robustness of the extraction method and its generalizability across different laboratories, irrespective of their geographical location.

### 2.4. Colorimetric and Spectroscopic Analysis of the Seeds

**Figure 6:**
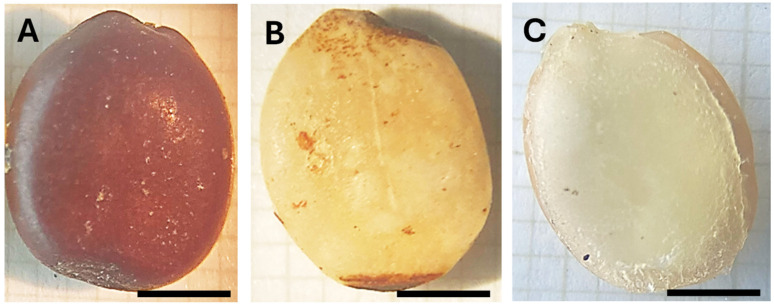
Images of a typical (**A**) pristine seed, (**B**) commercial dehusked seed (obtained from the company), and (**C**) dehusked seed obtained after applying our developed method under optimal conditions. The scale bars represent 0.3 cm.

At an industrial level, the success of the dehusking procedure is typically evaluated through a qualitative analysis. That is, if the obtained seeds are essentially white with no major signs of seed peel remains or degradation, the extraction conditions are considered suitable. Such an approach is often sufficient to fine-tune the process and perform minor adaptations in the extraction conditions if needed. However, this method is highly subjective and lacks robustness. In Figure 6, typical images of a pristine seed (with husk) and seeds dehusked by the company and by our method with optimal conditions are shown.

As illustrated in Figure 6B, the dehusked seeds obtained from the company (extraction performed solo using the H_2_SO_4_ as described in the introduction) exhibit minor brownish areas, indicative of incomplete peel removal. In contrast, the seeds obtained using our dehusking method (Figure 6C) demonstrate a complete absence of husk remains, underscoring the superior dehusking efficiency of the developed approach. To further validate these observations, a quantitative colorimetric assessment was conducted utilizing the CIELAB color space [18]. The L*, a*, and b* are represented in Table 4.

Clear differences in the seeds’ color parameters can be observed in Table 4. The lightness parameter, L*, exhibits the lowest value for the native seeds containing husk (A). In contrast, the seeds treated with the developed method (C) appear whiter, thereby reflecting light more efficiently and resulting in higher brightness. The presence of minor brown-dark regions in the endosperm (see Figure 6B) of the dehusked commercial seeds (B) places their L* values between those of the native seeds and the dehusked seeds obtained via the developed method.

Regarding the a* parameter (red-green axis), its value is always positive, being larger for the pristine seeds (A) since the brown color is expected to contain red components. The dehusked seeds (B and C) present no statistical difference with their values being low and close to zero, thus reflecting the minimal red/green influence.

Finally, the b* parameter (yellow-blue axis) is found to be always positive, with its maximum value being observed for the native seeds (A) (brown tones have yellow component), while the lowest value was observed for dehusked seeds by the developed method (C). The dehusked seeds from company (B) presented an intermediate value due to the yellowish tone of the endosperm and contributions from the minor brown areas where husk removal was not complete (see Figure 6).

**Figure 7:**
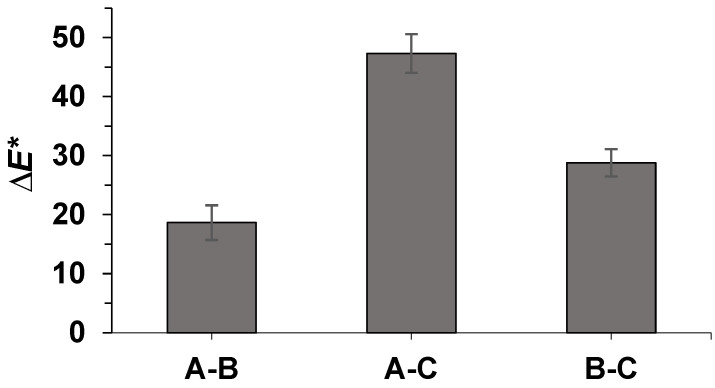
Color difference, $∆E^{*}$, among the different seed types (A: pristine seeds; B: dehusked seeds obtained with our optimal extraction method; C: commercial dehusked seeds from the company) as estimated using Equation (3).

The color difference $∆E^{*}$ (Equation (3)) among the three seed types is represented in Figure 7, where each seed pair comparison is represented in the *x*-axis. Two main observations can be highlighted: firstly, all seed comparisons display a ∆>3, which implies that the color differences are detectable by the human eye, as evidenced in Figure 6. Conversely, the maximum $∆E^{*}$ was observed among the native seeds and the dehusked seeds following the method developed in this study (i.e., collum A–C). This outcome validates the efficacy of the method in completely removing the seed husks, resulting in a great $∆E^{*}$.

**Figure 8:**
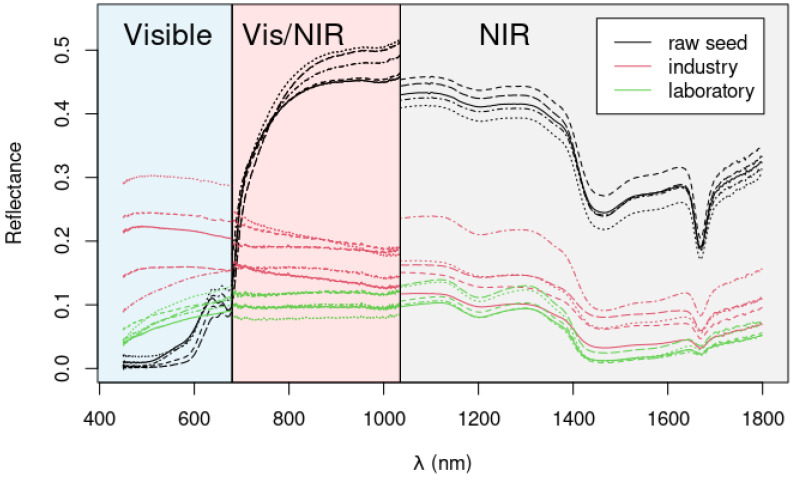
Diffuse reflectance spectra of pristine seeds (black curves), commercial dehusked seeds obtained with the industrial process (red curves), and dehusked seeds with our developed method (green curves). Five spectra per seed type are represented. The different regions of operation (i.e., visible, Vis/NIR, and NIR are highlighted).

The colorimetric analysis provides a simple and rapid method for assessing seed color differences and, consequently, evaluating the performance of the extraction method. However, if the extraction process affects the endosperm composition, these changes may not be reflected in the seed color. Therefore, a more robust analysis is required to determine the solvent’s impact. In this regard, diffuse reflectance spectroscopy was used for the first time to infer hypothetical changes in composition due to the extraction treatment. Four different spectrometers were used, operating in the visible and near-infrared bands (spectrometer A: 400–1000 nm, spectrometer B: 600–1130 nm, spectrometer C: 900–1700 nm and spectrometer D: 1000–2500 nm). Each spectrometer featured distinct internal configurations, diffraction gratings, etc., resulting in slightly different responses at the intersecting bands. This is shown in Figure 8, where the response of three spectrometers is depicted with different colors in the background to separate the corresponding spectra (five spectra per type of seed). The raw seeds (pristine seed) spectra are shown in black, whereas the spectra obtained from the industrially dehusked seeds are in red, and the spectra obtained from the seeds dehusked with our optimized method are shown in green. Clearly, the raw seeds absorb more in the visible due to the pigments and other molecules (anthocyanins, phenolic compounds, such as tannins and lignin). Conversely, the higher reflectance values observed in the near-infrared region are likely indicative of alterations in seed texture and firmness, a phenomenon frequently observed in other systems, including fruits [19,20]. A minor depression is evident at approximately 1200 nm, mainly related to the second overtone of the C-H stretching vibration, while a more pronounced depression is observed at 1430 nm, attributable to the first overtone of O-H stretching. An unusually strong dip around 1700 nm has been observed, but the origin of this remains to be elucidated. This coincides with the first overtone of C-H stretching in CH_2_ and CH_3_ groups (although this is typically a much larger feature). Overall, no major differences are noticed among dehusked seeds, which suggests that their composition is relatively similar and not affected by the extraction method.

**Figure 9:**
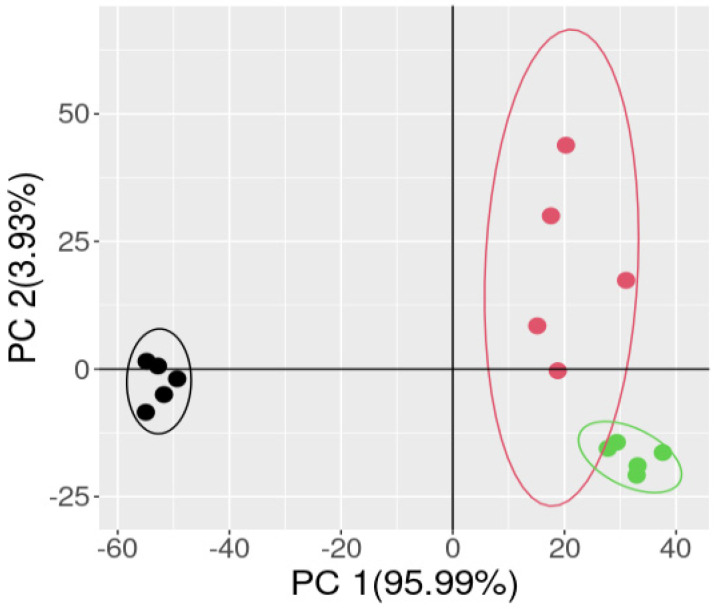
PCA analysis of the data where three clusters are highlighted, corresponding to the pristine seeds (black cluster), commercial dehusked seeds obtained with the industrial process (red cluster), and dehusked seeds with our developed method (green cluster).

The obtained spectra seem clearly separable, and a PCA plot of the first two scores of the data (Figure 9) show indeed a clear clustering of the samples. The raw seeds clearly separate from the processed ones along the first PC (which explains more than 99% of data variance). As can be observed, PC1 is the main responsible for discriminating in the space between the native seeds (negative scores) and the treated seeds (positive scores), showing the differences in physical characteristics between the treated and non-treated seeds. PC2 was more related to the differences between the industrial process (positive scores) and the developed method (negative scores). Consequently, it is demonstrated that there are differences in the developed method associated with the use of a greener approach.

## 3. Material and Methods

### 3.1. Plant Material and Chemicals

The company Industrial Farense, Lda (Faro, Portugal) kindly provided *Ceratonia siliqua* L. raw seeds as well as samples of dehusked seeds produced using their in-house standard H_2_SO_4_-based approach. The model seeds (i.e., dehusked seeds from the company) were used as a benchmark for comparison with the dehusked seeds obtained via the different extraction procedures developed in this work. The following solvents were used as received: formic acid (CH_2_O_2_, VWR Chemicals, Llinars del Vallé, Spain, 99–100%), acetic acid (C_2_H_4_O_2_, Sigma-Aldrich, Algés, Portugal, 99%), levulinic acid (C_5_H_8_O_3_, Across Organics, Madrid, Spain, 98%), methanesulfonic acid (CH_4_O_3_S Sigma-Aldrich 99%), hydrochloric acid (HCl, Supelco (Algés, Portugal, 37%) and sulfuric acid (H_2_SO_4_, Panreac, Barcelona, Spain, 96%).

### 3.2. Methods

#### 3.2.1. Seed Husk Extraction

The general process for extracting the carob seed husk involved adding a specific number of seeds (as received from the company) to 50 mL of solvent. Different extraction periods and temperatures, with constant stirring at 50 rpm, were tested. After extraction, the solvent mixture was removed, and the seeds were thoroughly washed for varying durations using different volumes of water. The seeds were then separated from the liquid phase and dried in an oven at 50 °C for 24 h to eliminate any residual moisture.

#### 3.2.2. Design of Experiments

The optimization of the extraction protocol was performed using an experimental design. Initially, the solvent effectiveness was evaluated using one-way ANOVA at a 95% confidence level. Subsequently, a Box–Behnken design coupled with response surface methodology (BBD-RSM) was employed, enabling the evaluation of the effects of individual parameters and their interactions on the response variables, utilizing a three-level design per factor: a lower level, an intermediate level, and an upper level. The Box–Behnken design is a more spherical experimental arrangement, avoiding axial points, which reduces the number of required experiments and prevents extreme conditions. In comparison to alternative experimental designs, this method minimizes resource consumption and saves time while maintaining robust analytical capability.

The variables selected were: (1) solid–liquid ratio (g seeds/50 mL of solvent; 3.5–17.75–25 g/50 mL); (2) Extraction temperature (60–75–80 °C); (3) % of alternative acid (70–80–100%); (4) contact time between the seeds and the solvent (5–7.5–10 min); (5) volume of water used for seed washing (50–75–100 mL); (6) washing time (5–7.5–10 min). The ranges were based on initial screening assays. The design yielded a total of 54 experiments, as detailed in Table 1.

The weight removed seed peel, *Y*, is given by Equation (1) and was used as a response variable.

(1) $$∆w=W_{initial}-W_{final}$$

where *W_initial_* represents the weight of raw seeds, and *W_final_* represents the weight of dry seeds after extraction. The estimated response value *Y* can be fitted to a polynomial equation of the second degree (Equation (2)), as follows:

(2) ∆*w* = *β*_0_ + *β*_1_X_1_ + *β*_2_X_2_ + *β*_3_X_3_ + *β*_4_X_4_ + *β*_5_X_5_ + *β*_6_X_6_ + *β*_12_X_1_X_2_ + *β*_13_X_1_X_3_ + *β*_14_X_1_X_4_ + *β*_15_X_1_X_5_ + *β*_16_X_1_X_6_ + *β*_23_X_2_X_3_ + *β*_24_X_2_X_4_ + *β*_25_X_2_X_5_ + *β*_26_X_2_X_6_ + *β*_34_X_3_X_4_ + *β*_35_X_3_X_5_ + *β*_36_X_3_X_6_ + *β*_45_X_4_X_5_ + *β*_46_X_4_X_6_ + *β*_56_X_5_X_6_+ *β*_11_X_1_^2^ + *β*_22_X_2_^2^ + *β*_33_X_3_^2^ + *β*_44_X_4_^2^ + *β*_55_X_5_^2^ + *β*_66_X_6_^2^

where *β*_0_ corresponds to the ordinate; X_1_ (solid–liquid ratio), X_2_ (extraction temperature), X_3_ (% of alternative acid), X_4_ (extraction time), X_5_ (volume of washing water), and X_6_ (washing time) are the independent variables; *β_i_* is the linear coefficients; *β_ij_* are the coefficients of the cross products; and *β_ii_* is the quadratic coefficient.

#### 3.2.3. Optical Microscopy

A professional-grade binocular microscope with achromatic optics (Olympus CH-2) was used for a preliminary visual analysis of the samples, including raw seeds, industrially processed seeds, and seeds dehusked using the different tested solvents and conditions.

#### 3.2.4. Colorimetric Analyzes

A Minolta CR-300 (Tokyo, Japan) tristimulus colorimeter was used to measure the color of the various carob seeds. A white standard porcelain plate (L*96.96; a*0.37; b*2.10) was used for calibration. The obtained color parameters were a* ranging from green to red, b* ranging from blue to yellow, and L* accounting for brightness (i.e., 0% for black and 100% for white), which translates to a numerical fluctuation from −60 to +60. The measurements were made three times at room temperature and with comparable lighting.

The total color difference between the samples was calculated according to Equation (3).

(3) $$∆E^{*}=\sqrt{{\left(∆L^{*}\right)}^{2}+{\left(∆a^{*}\right)}^{2}+{\left(∆b^{*}\right)}^{2}}$$

### 3.3. Diffuse Reflectance Spectroscopy

Diffuse reflectance spectra were acquired from the carob seeds in the range from 450 to 1800 nm. To optimize the signal, four spectrometers were used and their peak sensitivity spectral ranges were subsequently matched in the borders and concatenated. The spectral data between 450 and 680 nm (Visible, called henceforth by Vis band) were acquired using an Avantes AvaSpec-Mini2048CL-VI25 (full range 360–1100 nm) (Avantes, Apeldoorn, The Netherlands). The spectral data between 680 and 1035 nm (Visible-Near-Infrared, called henceforth by Vis/NIR band) were acquired using an Avantes AvaSpec HS2048XL-EVO (Avantes) (full range 600–1100 nm). The range 1035 to 1650 nm was obtained from an Avantes AvaSpecNIR256-1.7 TEC (Avantes) (full range 900–1700 nm). The fourth spectrometer, an Avantes AvaSpec NIR256 2.5 HSC EVO (Avantes) (full range 1000–2500 nm), allowed us to obtain information in the range 1650 to 1800 nm. The compound range 1035–1800 nm will be called henceforth by the NIR band. The range from 1800 to 2500 nm was too noisy and was discarded. The light source for the Vis and Vis/NIR bands measurements was a tungsten halogen AvaLight-HAL-S-Mini, while the NIR band data were acquired with a 150 W Philips PAR38 IR lamp (Avantes).

### 3.4. Statistical Analysis

A t-test with a 95% confidence level was performed to calculate the *p*-values for each variable studied. Variables with *p*-values less than 0.05 were considered significantly influential. The ANOVA and BBD-RSM design were generated and analyzed using the statistical software Statgraphics Centurion, version XVII (Statgraphics Technologies, Inc., The Plains, VA, USA). The spectroscopic analysis was performed in a script written by us [21]. The PCA analysis was performed through the R base function prcomp. The Savitzky-Golay filtering was performed through the function sgolayfilt of the signal package [22].

## 4. Conclusions

In this study, a range of acidic aqueous solvents were evaluated as potential alternatives to the current harsh industrial dehusking process of carob seeds, which relies on concentrated H_2_SO_4_. Among the tested solvents, CH_4_O_3_S was identified as the most promising, demonstrating reasonable performance (67% seed husk removal at 60 °C for 30 min). However, CH_2_O_2_ was also found to be effective, but led to irreversible seed damage. Consequently, the most promising solvent, CH_4_O_3_S, was selected for further optimization. This was achieved using a Box–Behnken design coupled with response surface methodology.

The optimal extraction conditions were determined to be 24.5 g of seeds in 50 mL of a solvent mixture containing 41% CH_4_O_3_S and 59% H_2_SO_4_, extracted at 90 °C for 10 min. This process yielded seeds that were morphologically robust and exhibited color characteristics comparable to, or superior to, commercially available seeds that had been dehusked with H_2_SO_4_. These findings indicate that CH_4_O_3_S is a viable and more environmentally friendly alternative to H_2_SO_4_.

A substantial decrease of a minimum of 40% in H_2_SO_4_ can be accomplished through the utilization of CH₄O₃, a method that is in accordance with the principles of green chemistry. CH_4_O_3_S has a reduced environmental impact: it does not generate harmful SO_x_ emissions, is less corrosive, more manageable, and readily biodegradable. These advantages position it as a prime candidate for industrial applications.

Another key finding of this study is the use of diffusive reflectance spectroscopy as a tool for characterizing seed features, which, to the best of our knowledge, has not been employed for this purpose before. The technique was found to be highly effective in distinguishing between treated and untreated seeds, providing valuable data on seed color and morphology with minimal sample preparation.

The optimized process has the potential for large-scale applications, representing a sustainable alternative that can significantly reduce the environmental footprint of the carob seed dehusking process. Subsequent research will concentrate on expanding the process, refining it further, and investigating its application in other industries that require environmentally friendly, efficient extraction processes.

## Figures and Tables

**Table 1:** BBD-RSM optimization using CH_4_O_3_S/H_2_SO_4_ mixtures.

Experiment	Solid–Liquid Ratio (g Seeds/50 mL Solvent)	Temperature (°C)	CH_4_O_3_S (%)	Extraction Time (min)	Volume of Water (mL)	Washing Time (min)	∆w (g)	∆w Adjusted (g)	Error (%)
1	2.5	75	70	7.5	75	10	0.34	0.34	1.81
2	13.75	60	80	7.5	50	5	0.52	0.52	0.45
3	2.5	75	80	5	50	7.5	0.22	0.23	4.00
4	25	75	80	5	50	7.5	0.88	0.84	4.09
5	13.75	75	100	5	75	10	1.07	0.99	6.85
6	13.75	85	80	7.5	50	10	3.14	3.37	7.46
7	13.75	60	100	7.5	50	7.5	2.74	2.76	0.72
8	13.75	75	80	7.5	75	7.5	2.16	2.32	7.42
9	13.75	90	80	7.5	100	10	2.28	2.12	7.07
10	25	75	100	7.5	75	10	1.09	1.18	8.51
11	13.75	75	70	5	75	10	1.56	1.50	4.07
12	13.75	60	70	7.5	100	7.5	0.54	0.53	0.75
13	13.75	75	80	7.5	75	7.5	1.98	1.92	3.19
14	13.75	75	70	10	75	5	2.04	2.01	1.52
15	2.5	75	80	10	100	7.5	0.35	0.35	0.86
16	13.75	90	80	7.5	50	5	2.00	1.92	3.73
17	2.5	60	80	5	75	7.5	0.09	0.10	5.78
18	25	60	80	10	75	7.5	2.53	2.52	0.19
19	25	90	80	10	75	10	8.86	8.84	0.18
20	13.75	60	80	7.5	100	5	0.67	0.61	8.70
21	13.75	90	100	7.5	50	7.5	2.86	2.70	5.57
22	13.75	90	70	7.5	50	7.5	2.07	2.02	2.11
23	13.75	75	70	5	75	5	1.54	1.60	3.80
24	13.75	75	100	10	75	10	1.12	1.19	6.00
25	2.5	60	80	10	75	7.5	0.26	0.25	3.69
26	25	75	80	10	100	7.5	3.64	3.38	7.27
27	2.5	75	100	7.5	75	5	0.21	0.21	2.08
28	13.75	75	80	7.5	75	7.5	1.93	2.12	9.93
29	2.5	75	80	5	100	7.5	0.27	0.27	2.36
30	13.75	75	80	7.5	75	7.5	2.34	2.32	0.88
31	25	90	100	5	75	7.5	1.83	1.99	8.70
32	2.5	75	100	7.5	75	10	0.30	0.29	0.76
33	25	75	80	10	50	7.5	4.98	5.02	0.75
34	13.75	60	70	7.5	50	7.5	1.67	1.83	9.94
35	25	75	70	7.5	75	10	2.88	2.95	2.49
36	25	60	80	5	75	7.5	2.22	2.00	10.10
37	2.5	75	70	7.5	75	5	0.52	0.53	1.03
38	13.75	75	80	7.5	75	7.5	2.75	2.32	15.61
39	13.75	75	80	7.5	75	7.5	2.76	2.32	15.92
40	25	85	100	10	75	8	5.86	5.19	11.39
41	13.75	75	100	10	75	5	2.94	3.08	4.69
42	13.75	60	100	7.5	100	7.5	1.96	1.64	16.32
43	13.75	90	70	7.5	100	7.5	3.52	3.56	1.24
44	13.75	60	80	7.5	100	10	1.82	1.76	3.41
45	13.75	90	80	7.5	100	5	3.15	3.35	6.43
46	25	75	70	7.5	75	5	5.39	4.52	16.20
47	25	75	50	5	100	7.5	3.45	3.47	0.79
48	13.75	90	100	7.5	100	7.5	4.12	3.51	14.63
49	13.75	90	80	7.5	50	10	3.47	3.30	5.13
50	2.5	90	80	10	75	7.5	0.75	0.75	0.70
51	2.5	75	80	10	50	7.5	0.53	0.53	0.44
52	2.5	90	80	5	75	7.5	0.46	0.41	9.94
53	13.75	75	100	5	75	5	2.59	2.17	16.46
54	13.75	75	70	10	75	10	2.47	2.68	8.13

**Table 2:** Results obtained from the ANOVA of BBD-RSM.

Variable	Sum of Squares	*F*-Value	*p*-Value
A: Solid–liquid ratio (g/50 mL)	64.366	68.49	0.0000
B: Temperature (°C)	12.308	13.10	0.0013
C: CH_4_O_3_S (%)	0.222	0.24	0.6309
D: Extraction time (min)	8.519	9.07	0.0057
E: Volume of water to wash (mL)	0.021	0.02	0.8844
F: Washing time (min)	1.449	1.54	0.2255
AA	0.125	0.13	0.7184
AB	3.238	3.45	0.0748
AC	0.115	0.12	0.7290
AD	7.250	7.72	0.0100
AE	0.228	0.24	0.6267
AF	6.477	6.89	0.0143
BB	0.345	0.37	0.5498
BC	0.152	0.16	0.6909
BD	5.847	6.22	0.0193
BE	2.057	2.19	0.1510
BF	1.248	1.33	0.2597
CC	0.073	0.08	0.7833
CD	0.129	0.14	0.7144
CE	0.003	0.00	0.9573
CF	2.095	2.23	0.1475
DD	0.723	0.77	0.3884
DE	2.133	2.27	0.1440
DF	0.002	0.00	0.9659
EE	0.237	0.25	0.6199
EF	1.818	1.94	0.1760
FF	0.490	0.52	0.4768
Error total	24.433		
Total (corr.)	146.330		

**Table 3:** Results obtained from repeatability and intermediate precision tests under optimal extraction conditions.

	Average (g)	Standard Deviation (g)	C.V. (%)
Repeatability (*n* = 9)	9.14	0.32	3.49
Intermediate Precision (*n* = 9)	10.08	0.82	4.12

**Table 4:** Color parameters for the (A) pristine seeds, (B) dehusked seeds obtained with our optimal extraction method, and (C) commercial dehusked seeds from the company.

Type of Seed	Color Parameters		
	L*	a*	b*
A	27.10 ± 2.57	4.76 ± 0.35	23.54 ± 2.73
B	43.16 ± 0.33	2.67 ± 0.87	14.28 ± 2.7
C	69.69 ± 2.16	2.08 ± 0.33	3.11 ± 1.28

## Data Availability

The original contributions presented in this study are included in the article. Further inquiries can be directed to the corresponding authors.
